# A Biosensor for Urea from Succinimide-Modified Acrylic Microspheres Based on Reflectance Transduction

**DOI:** 10.3390/s110908323

**Published:** 2011-08-26

**Authors:** Alizar Ulianas, Lee Yook Heng, Musa Ahmad

**Affiliations:** School of Chemical Sciences and Food Technology, Faculty of Science and Technology, University Kebangsaan Malaysia, Bangi, Selangor 43600, Malaysia; E-Mails: alizar_chem@yahoo.co.id (A.U.); andong@usim.edu.my (M.A.)

**Keywords:** urea, reflectance, optical biosensor, succinimide, acrylic microspheres, photopolymerisation

## Abstract

New acrylic microspheres were synthesised by photopolymerisation where the succinimide functional group was incorporated during the microsphere preparation. An optical biosensor for urea based on reflectance transduction with a large linear response range to urea was successfully developed using this material. The biosensor utilized succinimide-modified acrylic microspheres immobilized with a Nile blue chromoionophore (ETH 5294) for optical detection and urease enzyme was immobilized on the surface of the microspheres via the succinimide groups. No leaching of the enzyme or chromoionophore was observed. Hydrolysis of the urea by urease changes the pH and leads to a color change of the immobilized chromoionophore. When the color change was monitored by reflectance spectrophotometry, the linear response range of the biosensor to urea was from 0.01 to 1,000 mM (R^2^ = 0.97) with a limit of detection of 9.97 μM. The biosensor response showed good reproducibility (relative standard deviation = 1.43%, n = 5) with no interference by major cations such as Na^+^, K^+^, NH_4_^+^ and Mg^2+^. The use of reflectance as a transduction method led to a large linear response range that is better than that of many urea biosensors based on other optical transduction methods.

## 1. Introduction

Urea biosensors have been reported for urea determinations in biomedical, clinical and food industry applications [1–3], e.g., for monitoring of urea levels in human urine and blood for diagnosis of kidney function and health [4–6], as well as for the determination of urea levels in milk [2]. These numerous applications have motivated the development of urea biosensors. Urea biosensors have been developed using different transducers, including ion-selective field effect transistor electrode [7], ammonium ion-selective electrode [8], CO_2_ gas electrode [9], pH electrode [10] and ammonia gas selective electrode [11].

Many urease enzyme immobilization techniques for biosensor construction have involved the incorporation of the enzyme into a polymer membrane or polymer backbone such as tetrafluoroethylene and poly(tetrafluoroethylene) [1], chitosan membranes [12], sol-gel-derived thick-films [3,6] and poly(*N*-vinylcarbazole)/stearic acid [5]. Immobilization of urease in a membrane can lead to a loss of enzyme activity (deactivation and denaturation), as well as a decrease of the lifetime, sensitivity and poor response time of the biosensor. Therefore, it is essential to choose an enzyme immobilization technique that will enhance the operational and storage stability of the enzyme but at the same time avoid any interference of the immobilization matrix on enzyme activity [12–14]. Previously reported urease enzyme immobilization techniques include entrapment and encapsulation, covalent binding, cross-linking and adsorption [15]. Other published reports on the use of nanospheres for biosensors were based on poly-(styrene-*co*-acrylic acid) functionalized with dithioglycol [16,17] and poly(divinylbenzene-*co*-acrylic acid) [18] modified with gold nanoparticles for the immobilization of horseradish peroxidase enzyme. The use of spherical beads for immobilization of urease in biosensor is uncommon and no report in the use of acrylic microspheres for urea biosensor has been published to date. Nile-Blue chromoionophore is commonly used as an indicator in sol-gel film for biosensing of pesticides [19], in sol-gel [6] and in plasticized PVC membrane for biosensing of urea [20,21] with absorption optical detection mode.

In this research, acrylic microspheres modified with succinimide groups at the sphere surface were used to fabricate an optical urea biosensor based on a reflectance method. The microspheres serve two purposes, *i.e.*, first to covalently immobilize urease on the surface of the spheres and secondly to entrap the chromoionophore as an indicator in the spheres. *N*-Acryloxysuccinimide was used to modify acrylic microspheres to produce functional groups as a linker to immobilize enzyme urease via covalent bonds. The efficiency of this bonding has be proven as reported [22,23]. The acrylic microspheres have the advantages of small size and providing a large surface area to volume ratio for enzymatic reactions to occur on the surface, in addition to preventing any barriers to diffusion of reactants and products. Thus this should improve biosensor performance in terms of response time and linear response range. The use of *n*-butyl acrylate in microsphere synthesis is also compatible with the hydrophobicity of the lipophilic chromoionophore ETH5294 where the chromoionophore is used in the reflectance mode instead of the commonly used absorption mode. In addition, microspheres made from *n*-butyl acrylate also possess good adhesion properties that allow these spheres to be coated directly on a plastic substrate for optical biosensor fabrication.

## 2. Experimental

### 2.1. Chemicals

2-2-Dimethoxy-2-phenylacetophenone (DMPP) and 1,6-hexanadiol diacrylate (HDDA) were supplied by Aldrich. Sodium dodecyl sulfate (SDS), *N*-acryloxysuccinimide (NAS), urea, urease enzyme (E.C 3.5.1.5; 26.1 units/mg from Jack beans) and phenolphthalein (PP) were supplied by Systerm, Acros, Harnstoff, Sigma-Aldrich and Merck, respectively. Bradford Reagent and bovine serum albumin (BSA) were obtained from Sigma. Chromoionophores (ETH5294), MgCl_2_ and K_2_HPO_4_ were supplied by Fluka, while KH_2_PO_4_ and *n*-butyl acrylate (n-BA) were from Merck. NaCl, KCl and dimethylformamide (DMF) were obtained from Systerm, NH_4_Cl from Comak and 4-(*N-N*-dimethylamino)-benzaldehyde (DMAB) from Riedel de Haën. All aqueous solutions were prepared using deionized water.

### 2.2. Synthesis of Acrylic Microspheres

A mixture of 450 μL HDDA, 0.1 g DMPP, 6 mg NAS and 15 mL H_2_O with various amounts of n-BA and SDS was sonicated for 10 min, after which it was subjected to photopolymerisation for 600 s with UV light (350 nm) under nitrogen gas flow. The resulting acrylic microspheres were then collected by centrifugation at 4,000 rpm for 30 min. These spheres were washed three times in phosphate buffer at 0.05 M (pH 7.0), then air dried. The size of the acrylic microspheres was determined using scanning electron microscopy (SEM, LEO 1450VP). A Microtrac-X100 particle sizer was used to determine the size distribution of the acrylic microspheres (0.1 mg/mL). FTIR spectra of acrylic microsphere were obtained using a Spectrum FTIR GX infra-red spectrophotometer (Perkin Elmer).

### 2.3. Immobilization and Activity of Urease Enzyme

The acrylic microspheres were coated onto a transparent plastic sheet by dipping the plastic sheet in the acrylic microsphere suspension just after photocuring and air drying at room temperature. A solution of 2 mg of urease per mL of buffer was prepared in 0.05 M pH 7.0 phosphate buffer solution. The urease was then immobilized onto the acrylic microspheres by immersing the microsphere-coated support in the urease enzyme solution for 24 h at 4 °C. The acrylic microspheres with immobilized urease were then washed and kept in phosphate buffer solution at pH 7.0 until use. The plastic support with no immobilized acrylic microsphere coating was used for the control experiments.

The amount of urease immobilized on these samples was then estimated by checking the enzyme activity. For this measurement, 10 mL of test solution containing urea and phenolphthalein was prepared in deionized water and used for all measurements. Enzyme activity was determined by immersing a plastic support coated with a known amount of enzyme-modified microspheres in the urea solution. Urea hydrolysis by the immobilized urease on the microspheres produced a change in pH, which caused the test solution to turn pink. The enzyme urease catalyzes the urea hydrolysis to change pH according to the following reaction:

$$H_{2}N-\text{CO}-{\text{NH}}_{2}+{3H}_{2}O\overset{\text{Urease}}{\to }2{\text{NH}}_{4}{}^{+}+{\text{HCO}}_{3}{}^{-}+{\text{OH}}^{-}$$

The absorbance of the test solution was monitored for 30 min at a wavelength of 553 nm using the spectrophotometer.

### 2.4. Enzyme Leaching Test and Effects of pH on Enzyme Immobilization

To determine the effects of pH on the immobilization of the enzyme, urease enzyme solutions were prepared using phosphate buffer at pHs of 6.5, 7.0, 7.5 and 8.0. For the leaching test, a fixed amount of acrylic microspheres modified with immobilized urease were immersed in 0.05 M (pH 7.0) phosphate buffer solution, and 0.5 mL phosphate buffer solution was taken in every 10 min before mixing with 2.5 mL of urea solution (1.0 M) and phenolphthalein indicator. The mixture was monitored for 90 min, where the absorbance of that solution was periodically measured at a wavelength of 553 nm using the spectrophotometer.

### 2.5. Determination of Urease Immobilized

The amount of urease immobilized onto the urea biosensor and the influence of various immobilization times (0.5 to 24 h) were quantified by Bradford reagent method [24] with bovine serum albumin (BSA) as a standard to quantify urease enzyme immobilization. Urease enzyme concentration for immobilisation studies was fixed at 2.0 mg mL^−1^. The amount of immobilized enzyme was calculated according to the following equation:

% urease immobilized={(A−B)/A}×100%

where A is the amount of urease used and B is the urease left after immobilisation.

### 2.6. Construction and Evaluation of Urea Biosensor Performance

A sample of 375.0 mg of dried acrylic microspheres, 300.0 μL chromoionophore solution (0.1 mg mL^−1^ ethanol), 450.0 μL ethanol and 125.0 μL DMF were ultrasonically mixed for 3 min until homogeneous. After that 15.0 μL of the suspensions were drop-coated onto a fixed size plastic support (5.25 ± 0.01 mm diameter and 0.56 ± 0.05 mm thickness) and then dried at room temperature for 1 h. Urease was then immobilized onto these microspheres by dispensing 100 μL urease solution onto the support, then leaving it for 24 h at 4 °C. A urea biosensor in the form of a test-strip was thus fabricated and ready for testing.

To evaluate the response of the urea biosensor to urea, the biosensor was tested with several concentrations of urea solution in the 0.001 to 1,000 mM range in 0.05 M phosphate buffer (pH 6.5). The intensity of the color change of the urea biosensor was measured using a reflectance spectrophotometer (Mikropack DH-2000-Bal). Biosensors with varying amounts of acrylic microspheres (0.64, 6.43 and 643 mg) at different analysis times (5.0 min, 10 min and 20 min) were also evaluated. The linear response range of urea for the biosensor was determined from plots of urea concentration *versus* intensity change as measured by the reflectance spectrophotometer.

Since the enzymatic hydrolysis of the urea and the protonation of chromoionophore can be affected by pH, the working pH range of the optode was also optimized [20]. The effects of pH on the response of the urea biosensor was studied in phosphate buffer at pHs from 5.0 to 8.0 and with urea concentration fixed at 0.05 M.

### 2.7. Investigation of the Ionic Interference on the Optical Urea Biosensor

To determine the applicability of the optical urea biosensor to real samples, the interference of ions typically found in urine such as NH_4_^+^, Na^+^, K^+^ and Mg^2+^ was studied under optimum condition where the urea biosensor was exposed to the interference at various concentrations (0.001 to 100 mM).

### 2.8. Validation and Recovery Studies of Urea Biosensor

For validation and recovery performance of the urea biosensor, urine samples were used as described previously [6]. A non-enzymatic method using *p*-dimethylaminobenzaldehyde (DMAB) [25] was used as comparison. Briefly, for the chemical analysis of urea, DMAB solution was prepared in ethanol and HCl and the range of the standard urea concentration used was 1.0 to 70.0 mM to construct the calibration curve. For urine samples, urea concentrations of 1.01 to 69.94 mM were spiked into the samples. These spiked urine samples were analyzed for urea with both biosensor and DMAB methods. The percentage of recovery of urea from all urea-spiked urine samples was determined by the urea biosensor method, calculated with the following equation:

% Recovery=Cs/C×100%

where C is the actual concentration of urea and Cs is the concentration of urea determined by the biosensor in urea-spiked sample.

### 2.9. Reproducibility and Stability of the Urea Biosensor

The pH of the urea solution was kept constant during evaluation of the reproducibility and stability of the biosensor. Reproducibility of several urea biosensors using reflectance spectrophotometer were tested at a constant concentration of 10.0 mM urea at pH 6.5 (0.05 mM phosphate buffer). The constant reflectance response of each biosensor would indicate good reproducibility. Stability was determined by fabricating several urea biosensors, which were kept at 4 °C before used. Each biosensor was tested for up to 45 days using 1.0 mM and 10.0 mM urea in pH 6.5 (0.05 mM phosphate buffer).

## 3. Results and Discussion

### 3.1. Characterization of Acrylic Microspheres and Urease Immobilization

**Figure 1.**
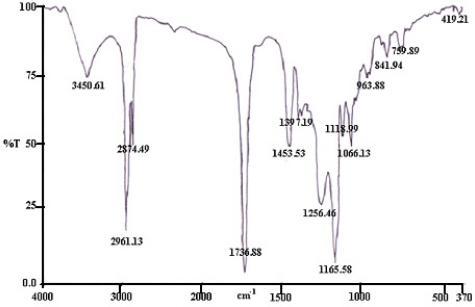
FTIR spectrum of acrylic microspheres.

In the FTIR spectrum of the acrylic microspheres (Figure 1), peaks at 1,188.99 cm^−1^ to 1,066.13 cm^−1^ are associated with the stretching frequencies of the (C–O) groups of the n-BA molecules and the succinimide ester (C–O) of the NAS molecules. The C–N stretch peaks present at 1,256.42 cm^−1^ to 1,165.58 cm^−1^ are from the NAS molecules.

Urease can be immobilized onto the surface of the acrylic microspheres by covalent binding because the NAS succinimide ester groups are highly reactive towards enzyme amino groups [22] and thus the amino groups of the urease.

**Figure 2.**
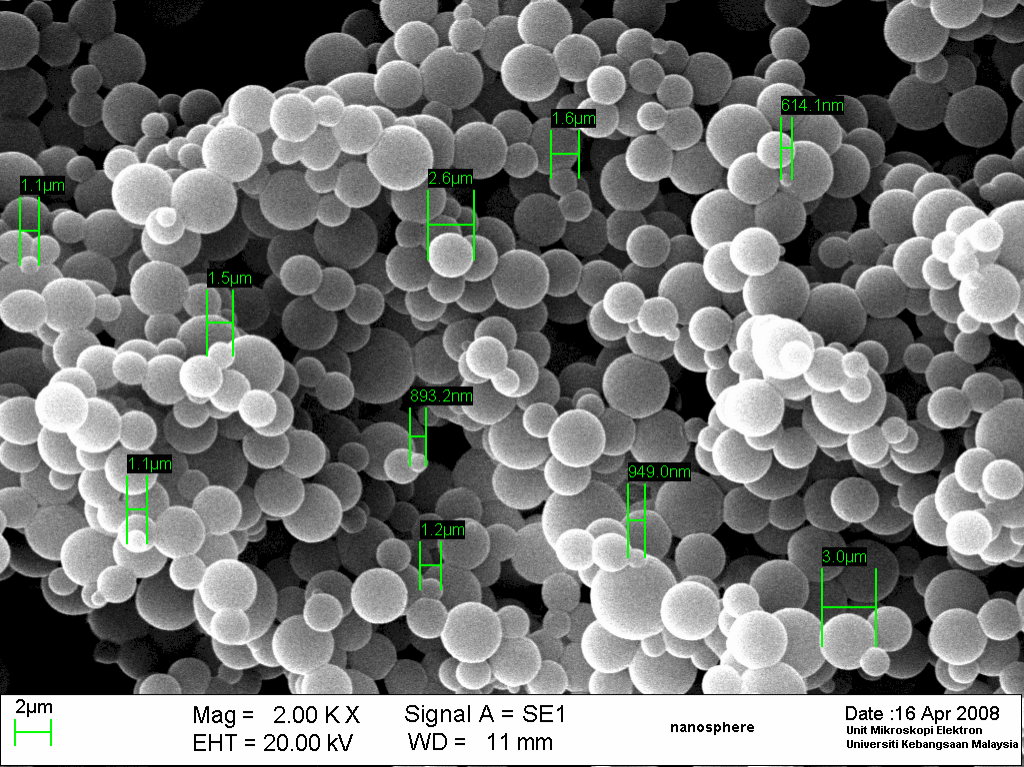
SEM image of acrylic microspheres prepared from photopolymerisation.

These spectra indicated that the acrylic microspheres had been modified with NAS. A typical scanning electron microscope (SEM) image of the acrylic microspheres (Figure 2) demonstrates that the size of the microspheres was approximately less than 5 μm in diameter, depending on the amount of monomer and SDS used. However, when the size of the microspheres was determined by a particle sizer, a size of more than 5 μm was predominant. This is because of aggregation of the acrylic spheres in solution phase during measurement when using a particle sizer.

Acrylic polymers with higher amounts of n-BA tend to be more sticky and result in microsphere aggregation [26]. The effect of the amount of n-BA monomer used on the size of the microspheres was more pronounced when compared with the variation of the amount of SDS used. Based on the particle sizer determination, with a fixed amount of SDS, an increase in the n-BA from 0.5 to 7 mL led to an increase in the size of the microspheres from 0.32 to 271 μm. This may be explained by the fact that a higher quantity of n-BA leads to the merging of the monomer into larger droplets and hence an increase in the size of the microspheres formed after photopolymerisation. Futhermore, after formation, microspheres with higher n-BA will aggregate more, a phenomenon attributable to the increased sticky properties during particle sizer measurements, where the microspheres appeared to have increased in size. The effect of the quantity of monomer on microsphere size has been reported previously [29] and the synthesis of acrylic microsphere by photopolymerisation in a suspension performed here behaved similarly. Although the SDS used is able to stabilize the monomer droplets to prevent formation of bigger droplets during emulsion polymerisation [27,28], an increase of SDS from 0.01 to 0.05 g produced microspheres of similar particle sizes. The size of the microsphere used for fabricating the biosensor consists is 1–5 μm based on the SEM images. The amount of n-BA monomer used to produce this microsphere composition was able to yield spheres that possessed a good adhesion characteristics, which is essential for coating onto a substrate for optical sensor.

**Figure 3.**
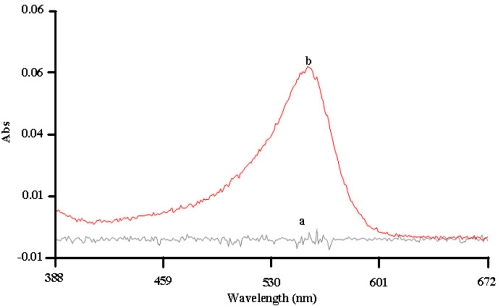
The absorbance response of the phenolphthalein indicator in the presence of acrylic microspheres and urea before immobilization with urease (**a**); and after immobilization (**b**).

The response of the immobilized urease was indicated by a change of colour of the phenolphthalein indicator to red in the presence of urea. The absorption spectrum of urea solution containing urease modified microspheres is shown in Figure 3. On the other hand, a plastic support coated with acrylic microspheres without immobilized urease showed no change in color when immersed in urea solution. This confirmed that the immobilized urease was still reactive where hydrolysis of urea by the urease producing NH_4_^+^, HCO_3_^−^, and OH^−^, which changed the pH towards alkaline values. Leaching experiments have shown that no leaching of urease enzyme that was immobilized on the acrylic microspheres occurred because a buffer solution exposed to the immobilised enzyme for 90 min did not change color when urea and the phenolphthalein indicator were added. This suggests that a very strong binding of urease with the succinimide ester groups of NAS on the acrylic microspheres.

### 3.2. Optimum pH for Enzyme Immobilization

**Figure 4.**
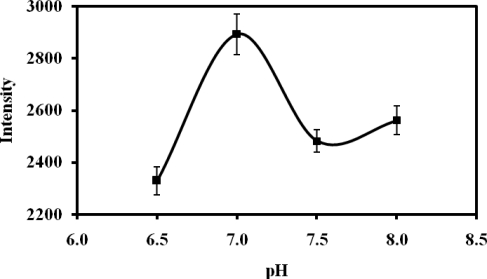
The reflectance intensity of the urease-modified microspheres prepared in the presence of different pH values (at 0.05 M phosphate buffer and 10 mM fixed urea concentration).

Optimization of pH conditions is essential for successful immobilization of the enzyme since a higher pH value will result in more amine groups being unprotonated and able to attack the succinimide ester group of the NAS moiety, but the ester is also susceptible to hydrolysis at high pH [22]. Figure 4 shows the effects of the use of different pHs for the enzyme immobilization step. The response of the biosensor increased with pH up to 7.0, after which it decreased with further increased in pH, indicating that pH 7.0 was optimum for suitable covalent binding between the amine groups of the enzyme with the succinimide ester of NAS. When the pH was higher than 7.0, which was outside the optimum working range of the enzyme urease, an overall decrease in the response of the measurement was thus observed. This finding is consistent with a previous report [22], which found that the amino group of urease directly reacted with the succinimide ester of NAS under the same pH.

### 3.3. Dependence of Biosensor Response on pH

**Figure 5.**
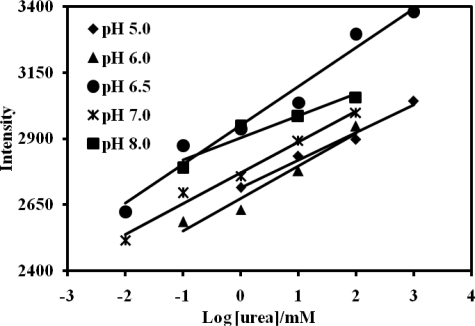
The effect of pH on the urea biosensor sensitivity.

Both the activity of urease and chromoionophore can be affected by pH of the buffer solution used, making it important to optimize the biosensor response with respect to pH. From the Figure 5, pH 6.5 was found to give the best response in terms of sensitivity and linear range for the urea biosensor (Table 1). This pH yielded a maximum sensitivity from the enzymatic response. Below this pH the histidine group of the urease enzyme is not affected by the phosphate buffer used [30].

### 3.4. The Effect of Loading of Acrylic Microspheres on the Response of the Urea Biosensor

**Figure 6.**
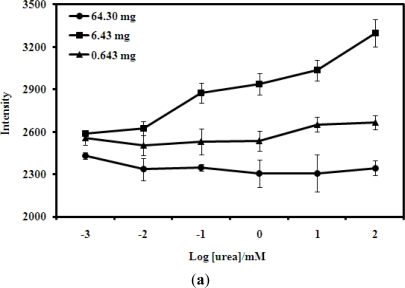

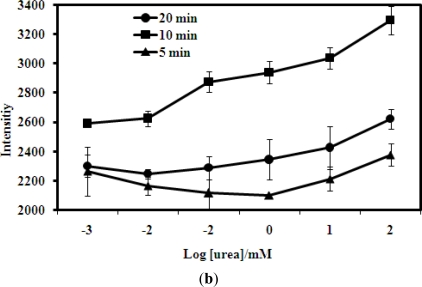
(**a**) The effects of the amount of acrylic microspheres coated onto a film on the urea biosensor response (measured at fixed time analysis 10 min); and (**b**) The effects of exposure time to urea on the response of the urea biosensor (fixed amount of coated microspheres).

Figure 6(a) shows the urea biosensor response with different amounts of acrylic microspheres coated onto a film. The response of the biosensor towards different amounts of microspheres indicated that the use of too few or too many microspheres would lead to poor responses. When too few microspheres were used, the intensity of the colour change will be low and thus the optode is insensitive, but when too many microspheres were loaded onto the film, a tight packing of these microspheres hindered the diffusion of the urea and reaction products. These factors eventually resulted in poor biosensor response. The response of the urea biosensor is best at 6.43 mg of acrylic microspheres for each coated film.

In terms of exposure time, the intensity of the colour change when the biosensor was exposed to urea was recorded after reaction times of 5, 10 and 20 min [Figure 6(b)]. When the intensity was taken at a short time of 5 min, little colour change was observed, indicating that the biosensor had not undergone sufficient reaction to produce products. On the other hand, when the exposure time was too long, the colour change was too dark and this resulted in small differences in reflectance changes. Therefore, exposure time of the biosensor to urea that would yield large changes in reflectance intensity was chosen at 10 min.

### 3.5. Effects of Ionic Interference on the Optical Urea Biosensor

**Figure 7.**
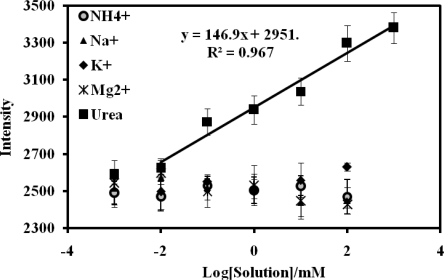
The effect of various ions on the response of the optical urea biosensor based on acrylic microspheres and reflectance transduction.

Acrylic polymer itself is not responsive to ions [26] whilst the chromoionophore ETH 5294 is sensitive only to pH [6,20,31]. Thus, above 0.01 mM urea, there was little response of the urea biosensor to the various ions used in the interference studies when compared to the response of urea (Figure 7). It indicates that these ions do not interfere with the urea response and the biosensor was selective to urea when used in the determination of urea in the urine samples.

### 3.6. Effect of Urease Immobilization Time Biosensor Response

**Figure 8.**
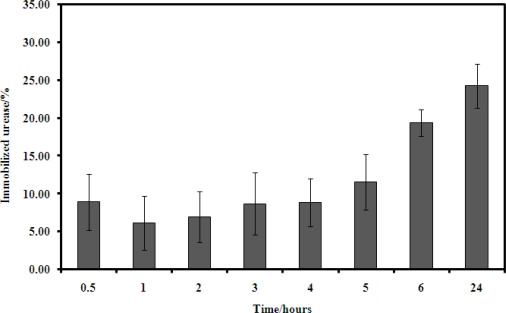
The amount of urease immobilized onto the urea biosensor at various immobilization times.

Figure 8 shows the amount of urease immobilized on the urea biosensor with the time employed during the process of urease immobilisation. The length of time for the immobilzation process affected the amount of enzyme immobilized onto the urea biosensor. Increasing in immobilization time obviously increased the amount of enzyme immobilized because binding of more enzyme via the succinimide ester could occur given a longer period of time. Thus, urease immobilisation on microspheres was performed over a 24 h period.

### 3.7. Validation and Recovery of Optical Urea Biosensor

**Figure 9.**
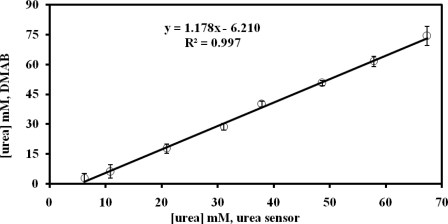
Urea concentrations in urine samples as determined by biosensor and DMAB.

Figure 9 correlates the results using the urea biosensor and the DMAB methods for urea determined in the urine sample. A good correlation coefficient (R^2^ = 0.997) and slope (1.18) for responses of the urea biosensor and DMAB methods to urea concentration was observed, indicating that the urea biosensor developed in this work can be used for the measurement of urea concentrations in urine samples.

Recovery by the urea biosensor is a very important indication of the accuracy of its detection of urea concentration in real samples of urine. Normally, the kidney excretes urea into the urine at 6 to 90 mg/dL (10.0 to 15.0 mM) [6]. Since the variation in level of urea in urine reflects the health of the kidney, therefore it is very important that the biosensor be able to determine the urea in urine very accurately. Table 2 shows the recovery of the optical urea biosensor containing different spiked urea concentrations in urine. The average recovery percentage of the urea biosensor across the concentrations of urea was 94.91% to 118.73% indicating no clear interference during determination of urea in urine samples by the optical biosensor.

### 3.8. Reproducibility and Stability of Urea Biosensor

**Figure 10.**
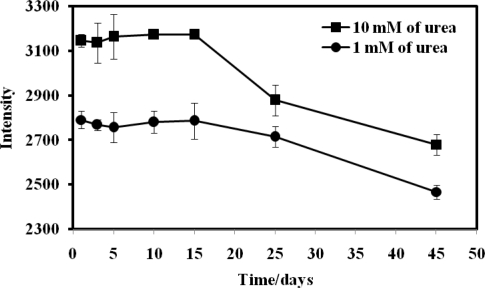
The stability of the urea biosensor response over a period of 45 days (in 0.05 M phosphate buffer solution at pH 7.0 containing 1.0 and 10.0 mM urea).

The reproducibility of the urea biosensor was satisfactory, with a small RSD of 1.43%. In terms of stability, the reflectance intensity of the biosensor was stable and remained at about 100% for the first 15 days and thereafter it was still remained at 87.01% and 82.37% of the initial intensity on day-1 for 1.0 mM and 10.0 mM urea, respectively, until the 45th day (Figure 10). The sensitivity of an optical urea biosensor based on sol-gel entrapment of urease enzyme was observed to decrease to 45% after 28 days storage [20]. The improvement in the long term stability of the optical urea biosensor based on acrylic microspheres was attributed to the covalent immobilization of the enzyme on the microsphere surface.

### 3.9. Comparison with Other Urea Biosensors

Table 3 presents a detailed comparison of optical urea biosensors reported in the literature with the biosensor reported in this work. By comparison, the urea biosensor presented in this work using acrylic microspheres as the immobilization matrix demonstrated a larger linear response range when compared to other types of optical based urea biosensors using various enzyme immobilization matrices. The acrylic microspheres based urea biosensor using reflectance transduction also compared favorably with other reported optical urea biosensors in terms of detection limit and response time.

## 4. Conclusions

An optical urea biosensor was successfully developed using acrylic microspheres as a matrix for urease immobilization. Urease enzyme can be immobilized onto the acrylic microspheres because of the introduction of NAS for enzyme binding onto the microsphere surface. In general the use of these microspheres coupled with reflectance transduction yielded a urea biosensor with an extended linear response range when compared with other reported optical urea biosensors, whilst maintaining similar detection limit, response time, selectivity and reproducibility. Besides that the optical urea biosensor is demonstrated to be suitable for measuring the concentration of urea in urine with good recovery when compared to the standard spectrophotometric method DMAB for the analysis of urine.

## Figures and Tables

**Table 1.** The effect of pH on the response of the urea biosensor based on acrylic microspheres and reflectance measurements (in 0.05 M phosphate buffer)

**pH**	**Sensitivity (I/mM)**	**R^2^**	**Linear range (mM)**
5.0	104.80	0.98	1.0–1,000
6.0	122.77	0.95	0.01–100
6.5	146.99	0.97	0.01–1,000
7.0	116.51	0.98	0.01–100
8.0	83.76	0.91	0.1–100

**Table 2.** Recovery performance of urea biosensor for urine samples spiked with urea at different concentrations (n = 3).

**Concentration of spiked urea (mM)**	**Concentration of urea as determined by urea biosensor (mM)**	**% of recovery by urea biosensor**
0	14.87	-
1.01	1.20	118.73
19.99	20.90	104.58
39.97	37.94	94.91
49.96	48.72	97.51
69.94	67.42	96.40

**Table 3.** A comparison of optical urea biosensor with previously reported urea biosensors.

**Urease immobilisation**	**Linear range (mM)**	**Limit of detection (mM)**	**Response time(min)**	**Reference**
Acrylic microspheres	0.01–1,000	0.01	10	This work
Sol-gel films	5–100	0.01	10	[6]
Poly(vinyl chloride) film	0.1–1	–	0.33	[20]
Sol-gel encapsulation	0.0025–0.05	0.0025	10	[32]
Alginate microspheres	0.1–60	0.1	8	[33]
Poly(vinyl chloride) membrane	0.3–100	0.3	3	[34]
Polypyrrole-polyvinyl sulphonate film	1–100	–	–	[35]
